# Isolation of a Novel Lytic Bacteriophage against a Nosocomial Methicillin-Resistant *Staphylococcus aureus* Belonging to ST45

**DOI:** 10.1155/2020/5463801

**Published:** 2020-12-22

**Authors:** Botond Zsombor Pertics, Dalma Szénásy, Dániel Dunai, Yannick Born, Lars Fieseler, Tamás Kovács, György Schneider

**Affiliations:** ^1^Department of Medical Microbiology and Immunology, Medical School, University of Pécs, Szigeti st. 12., H-7624 Pécs, Hungary; ^2^Institute of Food and Beverage Innovation, Food Microbiology, ZHAW School of Life Sciences and Facility Management, Einsiedlerstrasse 31, 8820 Wädenswil, Switzerland; ^3^Department of Biotechnology, Nanophagetherapy Center, Enviroinvest Corporation, Kertváros u. 2., H-7632 Pécs, Hungary

## Abstract

Methicillin-resistant *Staphylococcus aureus* (MRSA) can cause a wide range of infections from mild to life-threatening conditions. Its enhanced antibiotic resistance often leads to therapeutic failures and therefore alternative eradication methods must be considered. Potential candidates to control MRSA infections are bacteriophages and their lytic enzymes, lysins. In this study, we isolated a bacteriophage against a nosocomial MRSA strain belonging to the ST45 epidemiologic group. The phage belonging to *Caudovirales*, *Siphoviridae*, showed a narrow host range and stable lytic activity without the emergence of resistant MRSA clones. Phylogenetic analysis showed that the newly isolated *Staphylococcus* phage R4 belongs to the *Triavirus* genus in *Siphoviridae* family. Genetic analysis of the 45 kb sequence of R4 revealed 69 ORFs. No remnants of mobile genetic elements and traces of truncated genes were observed. We have localized the lysin (N-acetylmuramoyl-L-alanine amidase) gene of the new phage that was amplified, cloned, expressed, and purified. Its activity was verified by zymogram analysis. Our findings could potentially be used to develop specific anti-MRSA bacteriophage- and phage lysin-based therapeutic strategies against major clonal lineages and serotypes.

## 1. Introduction

*Staphylococcus aureus* is considered one of the most important pathogens, responsible for nosocomial infections affecting skin, respiratory system, bone, and soft tissues. These infections are frequently a challenging threat, due to the prevalent occurrence of antibiotic multidrug resistance among the isolates [1]. Methicillin-resistant *S. aureus* (MRSA) shows high risk to develop resistance to all available antibiotics in the near future [2].

Isolates have been categorized into three major groups, according to clinical and molecular epidemiology: (1) healthcare-associated (HA), (2) community-associated (CA), and (3) livestock-associated (LA) MRSAs. Distinction between these groups has become blurry because onset and subsequent infection routes can vary [3].

Multilocus sequence typing (MLST) is a widely accepted method for studying the origin, evolution, and clonal population structure of MRSA isolates [4]. With this method, several sequence types (ST) can be differentiated, including ST1, ST5, ST8, ST22, ST30, ST80, and ST239 which are the most frequently isolated global clones [5]. An emerging ST type is ST45, the closest relative of ST5 and ST22, and a major international representative of the epidemic MRSA (EMRSA) group [4]. ST45 was reported to be one predominant sequence type of HA-MRSAs and CA-MRSAs in Europe [6–9], North America [10, 11], Asia [12–16], and Australia [17]. This sequence type was associated with multiple serious infections [10, 18] and multiresistance to antibiotic therapy [10].

As antibiotic resistance is growing among the isolates, novel methods have to be seriously considered as potential therapeutic or preventive agents.

Bacteriophages (phages) are bacterial viruses that are able to kill the target bacterium cell. Unique features of bacteriophages allow them to recognize specific receptor structures on the surface of the bacterium cells, dock, and infect the host cell subsequently releasing phage progenies [19].

All known phages that infect *S. aureus* are the members of the *Caudovirales* order [20] and produce lysins, a group of evolutionarily advanced muralytic lytic enzymes that hydrolyze the bacterial cell wall peptidoglycan of bacteria [21, 22].

Application of bacteriophages against *S. aureus* has been implemented successfully in different models and in medicine. Their *in vivo* antibacterial effects and, by this, their raison d'etre in therapy were demonstrated in some recent studies [19, 23–26]. The ability of bacteria to develop resistance against certain phages [27], however, raises the need for prudent therapeutic approaches. Aside from the application of the ideal broad host range phages of *S. aureus*, narrow range phages must also be considered for full therapeutic coverage [28–31]. This could potentially eliminate the appearance of bacterial resistance and may increase the immediate antimicrobial effect, as theoretically more phage receptor types could be engaged by the different phages on the complex cell envelope structure of *S. aureus* [32, 33]. Using a library of multitudinous Staphylococcal phages with different receptor specificities enables us to target a broader range of bacterial strains, and with the help of narrow host range phages, a therapeutic broad host range effect can be gained. Thus, making phage libraries is an important strategy in the development of phage-based therapy.

Recombinant lytic phage enzymes such as endolysins or virion-associated lysins (VALs) are potential candidates for therapy, decolonization, and by this, infection prevention [34–36]. Lysins bind to and cleave highly conserved structures in the peptidoglycan layer that are highly immutable; thus, resistance against them is a rare event [21]. Therefore, recombinant endolysins are antimicrobial agents [37] with a high potential to treat Gram-positive bacterial infections such as MRSA [38, 39].

In this study, we isolated a novel lytic bacteriophage (R4), which was effective against a resident HA-MRSA isolate, originating from a German hospital and belonging to the international ST45 clonal group with growing epidemiological potential. We have identified, cloned, and expressed its cell wall degrading endolysin (amidase). Our results may aid the development of anti-MRSA bacteriophage-based therapeutic strategies targeting major clonal lineages.

## 2. Materials and Methods

### 2.1. Bacterial Strains and Growth Conditions

The clinical methicillin-resistant *Staphylococcus aureus* (MRSA) isolate 06-01019 (ST45) was isolated from the nostril of a hospitalized patient on selective mannitol salt agar. Species confirmation was performed by using Matrix-Assisted Laser Desorption/Ionization–Time of Flight Mass Spectrometry (MALDI-TOF MS) (Vitek MS, Biomerieux, Marcy-l'Étoile, France). The bacterium was routinely grown on lysogeny broth (LB) agar plates at 37°C or liquid LB medium (37°C at 125 rpm). To create a bacterial lawn, 100 *μ*l of the overnight (ON) liquid culture was plated onto a solid LB agar plate and incubated ON at 37°C. For amplification of bacteria and/or phages, ON cultures were poured into larger amounts of liquid medium and were incubated at 37°C in an orbital shaker.

### 2.2. Phage Isolation, Propagation, and Titer Determination

Bacteriophages were isolated from the local sewage farm (Pellérd, Hungary) with the traditional method [40]. Briefly, 1 ml of sewage sample was coincubated with the 50 ml midlog suspension of isolate 06-01019 ON at 37°C. The suspension was centrifuged (4,000 rpm, 10 min), and the supernatant was treated with chloroform in 1 : 50 *v*/*v* ratio (Molar Chemicals Kft., Halásztelek, Hungary) ON at 4°C. The presence of lytic phages was confirmed by spot testing [40] on the lawn of isolate 06-01019. A single phage plaque was cut out by using the agar overlay method [41] and was purified in three consecutive steps. The purified phage clone, named “Staphylococcus phage R4” according to the recent phage nomenclature [42], was propagated in 100 ml LB medium, centrifuged (11,000 rpm, 30 min), and resuspended in 50 ml deionized water (DW). The phage titer was obtained by serially diluting the phage suspension and subsequently spotting 10 microliters from the dilutions to get a countable amount of individual plaques on the lawn. Then, the number of plaque-forming units (PFU) was calculated for 1 ml of the concentrated suspension. The resulting high titer suspension (10^9^ PFU/ml) was used for further studies.

### 2.3. Host Range Determination and Phage Resistance Detection

Host range of R4 phage was determined by spot testing on 42 MRSA and 3 *S. aureus* strains from different collections. From the MRSAs, 14 HA-MRSA and CA-MRSA possessed known sequence types, as listed in Table 1. A clear spot indicated that the phage can kill the bacteria. Three distinct possible values were defined in terms of lytic efficacy, according to the clarity of the spot: fully cleared, partially cleared, and no clearance. Results were gathered as the mean of three different observations. The emergence of resistant mutants against R4 was tested on the lawn (2 × 10^8^/100 *μ*l) of the clinical methicillin-resistant *Staphylococcus aureus* (MRSA) isolate 06-01019 (ST45), by dropping 10 *μ*l dense phage suspension (10^9^ PFU/ml) on it and let it dry. This phage-covered area contained 10^7^ PFUs of R4 and around 3.5 × 10^6^ bacteria. After 24 hours of incubation, lack or emergence of phage resistant clones was visually detected.

### 2.4. Transmission Electron Microscopy (TEM)

Morphology of R4 phage was performed by TEM as described recently [43]. Briefly, 10 *μ*l from the purified high titer (10^9^ PFU/ml) phage stock was deposited onto formvar-coated copper grids (Pelco Grids, Redding, Canada) and negatively stained with 1.5% *w*/*v* phospho-tungstic acid (Merck KGaA, Darmstadt, Germany) for 40 seconds. After drying, phages were visualized on a JEM-1400 Flash TEM (JEOL USA Inc., Peabody, USA) transmission electron microscope operated at 80 kV acceleration voltage, with 54 *μ*A beam current.

### 2.5. Phage Genome Sequence Determination and Bioinformatic Analysis

Phage DNA was isolated from phage stocks with a concentration ≥109 PFU/ml. Phage DNA was extracted and purified based on the recently described method [43]. A phage lysate from the high titer phage suspension was obtained by using a QIAGEN Lambda Midi Kit (QIAGEN Inc., California, USA) and following the manufacturer's protocol. The purified phage DNA was dissolved in 100 *μ*l of sterile nuclease-free H_2_O and was used to prepare Genomic DNA sequencing libraries by using the Nextera XT Library Preparation kit (Illumina, California, USA). Sequencing was performed using MiSeq Reagent Kit v2 (2 × 150 bp) on an Illumina MiSeq instrument (Illumina, California, USA). The Mypro pipeline was used to assemble the gained pure sequences.

The assembled sequence was annotated on the RAST server (https://rast.theseed.org/FIG/rast.cgi). CLC Sequence Viewer v.6 (CLC bio, Aarhus, Denmark) was used to analyze the annotated genome and to illustrate the genome map. Open reading frames (ORFs) and gene predictions were confirmed by GeneMarkS program [44]. Homology searches were conducted by the BLAST tools available at NCBI website (https://www.ncbi.nlm.nih.gov/blast). R4 phage was classified according to the guidelines of the International Committee on Taxonomy of Viruses (ICTV, http://talk.ictvonline.org/taxonomy/) supported by ViralZone (http://viralzone.expasy.org) and BLASTn results. Protein homology prediction was conducted by NCBI BLASTp and HHpred tool of MPI Bioinformatics Toolkit (http://toolkit.tuebingen.mpg.de/tools/hhpred).

The nucleotide sequence of R4 was deposited in the GenBank database under the accession number MT366568.

### 2.6. Phylogenetic Analysis of R4 Phage

Whole genome-based phylogenetic analysis was conducted with VICTOR [45], involving the first 27 highly similar siphophages, according to the homology searches. All pairwise comparisons of the nucleotide sequence were conducted using the Genome-BLAST Distance Phylogeny (GBDP) method, under settings recommended for prokaryotic viruses. Branch support was inferred from 100 pseudobootstrap replicates each. Tree was rooted at the midpoint [46] and visualized with FigTree [47]. Taxon boundaries at the species, genus, and family level were estimated with the OPTSIL program, with the recommended clustering thresholds and an *F* value (fraction of links required for cluster fusion) of 0.5 [48].

### 2.7. Cloning of the Endolysin Gene

The designed primer pairs containing overhanging ends with *Xho*I and *Hind*III enzyme recognition sites (5′-TAAATGTTA**CTCGAG**ATGTTGATAACAAAAAACCAAGCGAAAAA-3′ and 5′-TAAATGTTA**AAGCTT**CTAAATCGTGCTAAACTTACCAAAACTACT-3′ (Sigma-Aldrich, St. Louis, USA)) were used to amplify *orf27*, coding for an endolysin (amidase) from the purified DNA of R4 phage. The PCR reaction was performed by using the Pfu DNA polymerase enzyme mix (Thermo Fisher Scientific, Waltham, USA) with the following conditions: denaturation at 95°C for 2 min, followed by 30 cycles, consisting of 95°C and 30 seconds denaturation, 54°C and 30 seconds annealing, and 72°C and 1 min elongation. The amplification was ended by an additional 10 min 72°C postelongation phase. The amplified PCR fragment was separated (1% agarose gel), extracted (Gel Extraction Kit; Thermo Fisher Scientific, Waltham, USA), and blunt-end cloned (ON, 20°C) into the linearized pJET1.2/blunt plasmid by using the Clone JET PCR Cloning Kit (Thermo Fisher Scientific, Waltham, USA). The ligation mixture was heat-shock transformed into the laboratory *Escherichia coli* strain XL1-Blue. The integrated PCR insert was double digested with *Xho*I and *Hind*III (Thermo Fisher Scientific, Waltham, USA), and after separation (1% agarose gel) and the consecutive purification (Gel Extraction Kit) step, it was religated into the *Xho*I and *Hind*III linearized pRSET A expression vector (Thermo Fisher Scientific, Waltham, USA). Ligated constructs were ethanol precipitated and, after resuspension in DW, were electroporated into the laboratory *E. coli* strains DH5*α* and BL21 by using 1 mm diameter cuvettes and the GenePulser XCell system (Bio-Rad, Hercules, USA) with 1.8 kV voltage and 600*Ω* resistance. Transformed cells were selected on ampicillin-containing (100*μ*g/ml) LB agar plates incubated ON at 37°C. Integration and presence of the resulting *orf27*-pRSET A construct was confirmed with the double digestion of the isolated plasmids and with PCR.

### 2.8. Protein Expression, 1 Dimensional Gel-Electrophoresis

5 ml ON cultures of (1) BL21, (2) BL21 with pRSET A, and (3) BL21 containing the *orf27*-pRSET A plasmid construct were used to start log phase (OD_600_=0.8) cultures and were further incubated without (control) and with isopropyl-*β*-D-thiogalactoside (IPTG) reaching 2 mM final concentration. From the resulting cultures, 1 ml samples were taken at 0, 1, 2, 3, 4, and 5 h timepoints. Samples were centrifuged, and the pellets were resuspended either in distilled water boiling at 100°C for 10 min, or in sonication buffer (pH 7.4, 50 mM Tris, 1 mM EDTA) for sonication. Sonication was performed four cycles of 0.5-0.5 seconds of pulsation for 1 min at 25-30% amplitude. After each cycle, there was a 1-2 min incubation period on ice. Boiled or sonicated samples were mixed with 1 : 4 (*v*/*v*) volume of 5x Sample Buffer (0.6 ml 1 M Tris pH 6.8, 5 ml 50% glycerol, 2 ml 10% sodium dodecyl sulfate (SDS), 0.5 ml *β*-mercaptoethanol, bromophenol blue, filled with distilled water up to 10 ml) and electrophoresed on a 10% polyacrylamide gel [49] for 1.5 h at 120 V, using the Bio-Rad Mini Protean II system (Bio-Rad, Hercules, USA). After separation, bands were visualized with Coomassie Brillant Blue (R-250, Reanal, Budapest, Hungary) staining [50].

Polyhistidine tag-based affinity purification of the expressed endolysin was performed from 10 ml log phase culture that was induced with IPTG (2 mM) for 5 h. The centrifuged pellet was resuspended in His-binding buffer and was sonicated as described above. Purification of the endolysin was implemented with the His-Spin Protein Miniprep (Zymo Research, Irvine, USA), according to the protocol. The purified protein was also visualized by polyacrylamide gel electrophoresis.

### 2.9. Zymography

Zymography analysis was carried out as previously described with modifications [51]. Briefly, *S. aureus* isolate 06-01019 (ST45) was grown and collected from 200 ml of log phase culture. After washing the pellet with 25 ml lysis buffer A (pH 6.2, 50 mM ammonium acetate, 10 mM CaCl_2_, 1 mM dithiothreitol), the pellet was resuspended in 1.5 ml lysis buffer A. Of this, 500*μ*l was added to 3 ml lysis buffer A, resulting in a solution of OD_600_=~10. From this dense bacterial suspension, 500 *μ*l was incorporated into 5 ml total volume of 10% polyacrylamide separating gel (1.665 ml acrylamide/bis-acrylamide, 1.25 ml 1.5 M Tris (pH 8.8), 5 *μ*l 10% SDS, 1.7 ml distilled water, 35*μ*l 10% ammonium persulfate and 2.5 *μ*l TEMED), which was casted subsequently. Sonicated and purified samples were electrophoresed (120 V, 1.5 h). The gel was then washed with DW for 30 min, and with renaturation buffer (pH 6.9, 25 mM Tris-HCl, 10 mM MgCl_2_, 0.1% Triton X-100) at 37°C ON. The gel was stained for 3 h in 0.1% methylene blue containing 0.01% KOH and destained with DW until the cleared zones were visible.

## 3. Results

### 3.1. Morphological Features of R4 Phage

**Figure 1:**
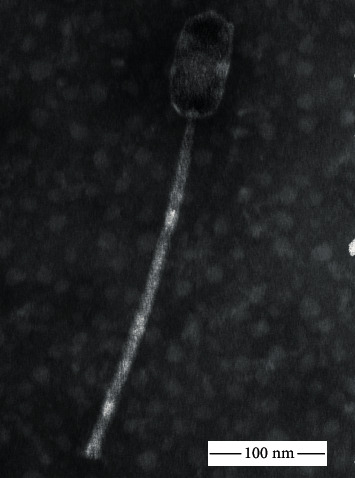
Transmission electron microscopic picture of phage R4 showed the typical characteristic of the *Siphoviridae* family, with an elongated head.

R4 is a tailed phage with an approximately 100 nm long and 50 nm wide prolate head. The flexible tail is about 300 nm long (Figure 1). This morphology is a characteristic of the members of the *Triavirus* genus, *Siphoviridae* family. The phage produces relatively small individual plaques with a diameter of 0.5 mm on the host strain lawn after overnight incubation at 37°C.

### 3.2. Host Spectrum of R4 Phage and Phage Resistance Testing

The results of host spectrum determination can be seen in Table 1. Out of 45 examined strains (including the host), 14 seemed to be susceptible on different levels to the R4 phage, and 31 were totally resistant. Besides its host strain (06-01019), phage R4 showed a strong lytic effect on one HA-MRSA strain (ST254) and one CA-MRSA (ST1) (Table 1). A partial lytic effect was revealed against three CA-MRSA strains (ST5, ST8, and ST22). Out of 19 more tested MRSA isolates from another collection, 8 were susceptible and showed full lysis, while 11 were resistant. The remaining 9 MRSA and the 3 *S. aureus* from different collections showed no sensitivity to the R4 phage.

No emergence of resistant clones was detected during the study on the lawn of the clinical methicillin-resistant *Staphylococcus aureus* (MRSA) isolate 06-01019 (ST45).

### 3.3. Genomic Properties of R4 Phage

Basic genome statistics revealed that R4 phage has a 27.902 MDa double-stranded, linear DNA with a length of 45 168 base-pairs and a G+C content of 33.3% (37.4% adenine, 13.7% cytosine, 19.6% guanine, 29.3% thymine).

The genome shows homology with *Staphylococcus* phage SAP8 (MK801680.1), SMSAP5 (JQ779023.1), SH-St 15644 (MG770897.1), and vB_SauS_fPfSau02 (MK348510.1) with coverage of 90%, 90%, 85%, and 71% and sequence identity of 97.18%, 97.18%, 97.59%, and 96.87%, respectively, and also with chromosomes of different *S. aureus* strains, such as 2395 USA500 (CP007499.1) and AR_0468 (CP029657.1) with coverage of 94% and 88%, and sequence identity of 99.22% and 99.19%, respectively. Besides the previously mentioned four siphophages, the genome shows homology (on different levels) with phages classified as members of the *Triavirus* genus, such as *Staphylococcus virus* IPLA35 (EU861005.1), phiSLT (AB045978.2), phi12 (AF424782.1), 3a (AY954956.1), 42e (AY954955.1), and 47 (AY954957.1). This is consistent with the morphological properties and consolidates that the R4 phage belongs to the *Triavirus* genus in the *Siphoviridae* family.

**Figure 2:**
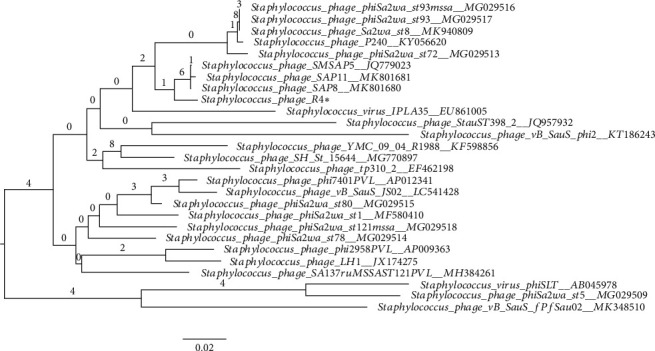
Phylogeny tree of R4 phage. Genome-BLAST Distance Phylogeny (GBDP) tree inferred using the formula D0 and yielding average support of 2%. The numbers above branches are GBDP pseudobootstrap support values from 100 replications. The branch lengths of the resulting VICTOR trees are scaled in terms of the respective distance formula used. The OPTSIL clustering yielded twenty species clusters, one cluster at the genus level, and one at the family level. Accession numbers are indicated next to the phage names. R4 phage is marked with a black asterisk.

Figure 2 shows the phylogenomic relations of the R4 phage with other Staphylococcal phages and reveals the closest relation to phage SAP8, SAP11, and SMSAP5.

**Figure 3:**
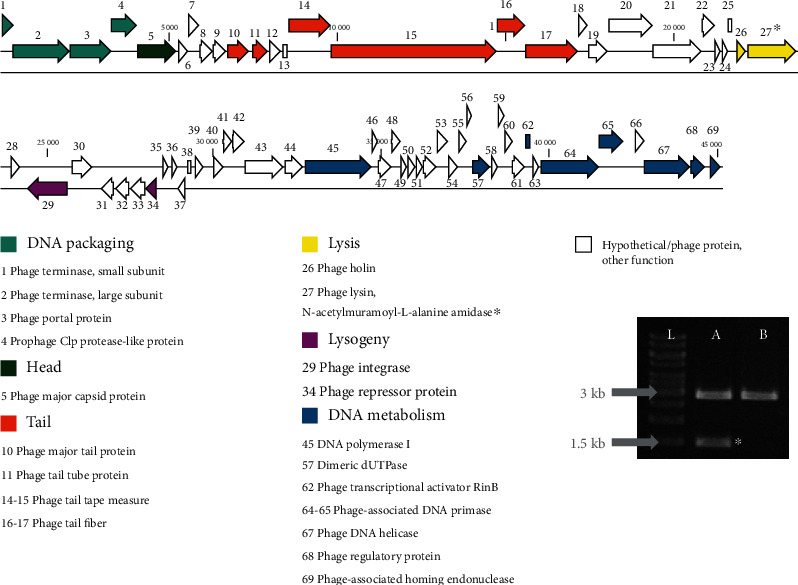
Genome organization of the R4 phage. The 45 kb double-stranded DNA (black parallel lines) contains 69 coding genes, each represented by arrows, annotated as indicated. The modular genome organization, characteristic for Staphylococcal siphophages is highlighted by colors, indicating the coding regions belonging to one of the six functional modules. The 1455 bp long gene *orf27*, coding for the endolysin (amidase), marked by a black asterisk, was cloned into the 2.9 kb pRSET A vector. The rightmost black image represents the result of *Xho*I-*Hind*III probe digestion of *orf27*-pRSET A construct (lane “A”) and pRSET A control (lane “B”) on 1% agarose gel. Excised 1.5 kb *orf27* band is marked by a white asterisk. (Lane “L”: DNA ladder.)

Annotation resulted in 69 and 70 protein-coding sequences by RAST and GeneMarkS, respectively (Figure 3). Functions of certain genes were predicted accordingly by all platforms. The genome is showing the modularity, characteristic for Staphylococcal *Siphoviridae*, having six functional modules: DNA packaging, head structure, tail structure, lysis, lysogeny, and DNA replication/metabolism. Although genes accountable for integration are present, no remnants of mobile genetic elements and traces of truncated genes were observed.

### 3.4. Molecular Properties of the R4 Phage Endolysin, R4lys

The genome of R4 contains a lysis cassette, consisting of a lysin and a holin (Figure 3, yellow arrows, gene 26 and 27). The 1455 bp long *orf27* (from nucleotide 22 176 to 23 630) coding for the 484 amino acid long R4lys endolysin shows strong similarity (>96%) with the corresponding gene segment of *Staphylococcus* siphophages YMC/09/04/R1988 (KF598856.1), phi12 (AF424782.1), 47 (AY954957.1), LH1 (JX174275.1) of the *Triavirus* genus, siphophages 96 (AY954960.1), TEM123 (JQ779024.1) and TEM126 (HQ127381.1) of *Phietavirus* genus, and many other *Staphylococcus* siphophages, each coding a muralytic peptide. The endolysin of R4 phage itself was predicted to be an N-acetylmuramoyl-L-alanine amidase with two putative conserved domains: an amidase-3 (pfam01520) in the middle (aa residues 181-362), and a SH3b (cell wall binding) domain at the C-terminal (aa residues 402-470). Therefore, the coding gene, *orf27*, was selected for amplification and cloning (Figure 3) and for expression of the R4lys endolysin of R4 phage.

### 3.5. Protein Expression and Amidase Activity of R4lys

**Figure 4:**
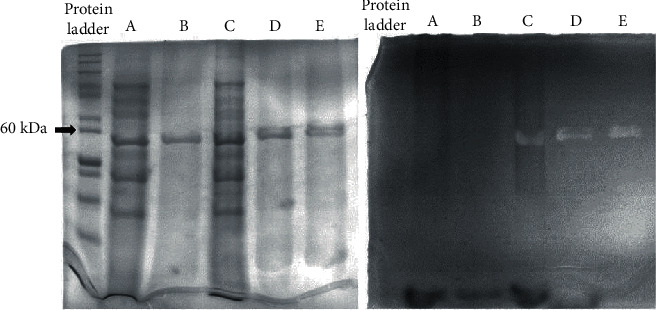
Visualization of the expressed recombinant R4lys endolysin. Comparison of sonicated and boiled crude expression cell lysates and purified amidase by SDS-PAGE (left) and by zymogram (right) analysis. The letters are indicating that the same samples were added to both gels in the corresponding lanes. Expressing BL21 cells were harboring the *orf27*-pRSET A plasmid. (a) Sonicated crude BL21 cells, boiled, with 5x sample buffer. (b) Column purified endolysin, boiled, with 5x sample buffer. (c) Sonicated crude BL21 cells, not boiled, with 5x sample buffer. (d) Column purified endolysin, not boiled, with 5x sample buffer. (e) Column purified endolysin, not boiled, without 5x sample buffer. SDS-PAGE revealed the presence of a protein, slightly below 60 kDa (black arrow is showing 60 kDa on the protein ladder), regardless of the sample preparation method. In spite of the R4lys protein's presence, zymography only showed its cell wall destroying amidase activity when the sample was not boiled (white bands in lanes (c, d, e)).

SDS-PAGE analysis revealed the successful overexpression of R4lys endolysin (Figure 4). Sole enhancement of a particular band below 60 kDa only in lanes with *orf27*-pRSET A plasmid construct-containing BL21 lysates suggests the presence of the overexpressed, target protein. In a time-course experiment, it was observed that IPTG boosts the expression as time passes (data not shown). However, overall results showed that protein expression was observable even without IPTG addition, and there is no remarkable difference between expression levels of ON IPTG-treated and untreated samples.

After purification of the R4lys, strong bands appeared on the gel exclusively close to the 60 kDa protein ladder band, also indicating the presence of the target protein. The R4lys was not traceable with SDS-PAGE analysis from the supernatant of the centrifuged ON cultures before cell lysis, even by different protein precipitation methods (data not shown). It seems that the protein is detained in inclusion bodies of BL21 cells and not transported to the external environment.

A difference was not visible between boiled and sonicated samples, regarding band intensity on the protein gel. However, boiling abolished the enzymatic activity; thus, no clearing zones could be observed on the zymogram gel (Figure 4). In contrast, in the absence of heat treatment, clearing zones in the gel were clearly observed due to the lysis of the target bacterium. Other proteins in the protein ladder or in the sonicated BL21 did not create clearing zones, and by this, the amidase enzyme activity of R4lys was confirmed.

## 4. Discussion

In this study, we have successfully isolated and characterized a novel *Staphylococcus* phage, R4, a new member of the *Triavirus* genus in *Siphoviridae*. We have cloned and expressed the R4lys amidase of the phage and showed that the protein possesses lytic activity against the peptidoglycan of the investigated host strain. With a functional assay, we have identified the amidase gene that encoded the enzymatically active enzyme and that can cause the lysis of the target cells.

According to a proposed classification, Staphylococcal phages fall into three distinctive categories, determined by their genome size. Class I phages with the smallest genome (<20 kb) are podoviruses, class II with average genome size (~40 kb) are siphoviruses, and class III is for myoviruses with the largest genome (>125 kb) [20, 52, 53]. The 45 kb genome size and the morphological properties of R4 phage suggest that it belongs to class II of Staphylococcal phages. This is confirmed by the modular structure of the R4 genome (Figure 3), which is also a phenomenon, specific for this class [53]. While Staphylococcal phages of the *Myoviridae* family usually have a broad host range and lytic properties, making them the best candidates for phage therapy purposes [33, 53–56], siphoviruses are often temperate as they harbor a lysogeny module (Figure 3, purple). This implicates concerns of their use as therapeutic agents, due to the integration into the host genome as a prophage without causing lysis and to potential horizontal gene transfer between bacterial genomes. Remarkably, certain *S. aureus* virulence factors are encoded by Staphylococcal prophages [20, 52, 53, 57]. Despite this fact, it is experimentally proven that Staphylococcal temperate siphoviruses can also be effective as antimicrobials against MRSA in mouse models [23, 24], with a mutated lysogeny module [58, 59]. Spot tests and TEM images (Figure 1) confirmed that R4 is indeed a lytic phage.

By focusing on the application of phage-derived lytic proteins, we can exclude the concerns with phage therapy in general. Besides resistance, for example, even obligatory lytic phages have many proteins with hypothetical or unknown function. Properties (structure, pharmacokinetics, etc.) of a recombinant endolysin can be well-defined and better controlled during the production of an antibacterial agent, in terms of manufacturing a pure agent with detailed, uniform composition [60].

Modularity is prevalent in Staphylococcal lysins, usually consisting of 2 enzymatically active (amidase and CHAP) and a cell wall binding SH3 conserved domain [21, 35, 36, 61]. The recently published LysSAP8 endolysin of SAP8 phage followed this trait [62] and showed promising properties for use as an antibacterial agent. Our results revealed that the novel R4 phage and its R4lys endolysin are closely related to SAP8 and LysSAP8, respectively. Homology search results did not show a CHAP domain to be highly similar with the same conserved segment of homologous proteins on the N′-terminal. Nevertheless, R4lys endolysin is a modular protein with 2 of the conserved domains. Combining different lysins with different cleavage sites and applying them at the same time often results in synergy [21]. Synergistic activity can be also achieved by using a lysin with certain antibiotics. This can be further enhanced by creating chimeric fusion proteins [61] by combining different domains with different cell wall recognition specificities. Our results could contribute to the development of these techniques.

## 5. Conclusions

Our results suggest that the R4 phage and its endolysin, R4lys, can be promising candidates for further investigation in phage-research.

As this phage targeted a high-risk international clone, our results may aid the development of bacteriophage-based therapeutic strategies for MRSA and/or VRSA strains, targeting major clonal lineages.

## Figures and Tables

**Table 1:** Host range of the R4 phage. Bold and italic fonts indicate the full and partial lysis of the strain by R4, respectively. Host strain of phage R4 is marked with an asterisk.

Species	Strain code/origin	Sequence type	R4 phage effect
HA-MRSA	06-01388	ST247	No lysis
	**93-01000**	**ST254**	**Full lysis**
	06-01597	ST239	No lysis
	06-02182	ST22	No lysis
	**06-01019**	**ST45**	**Full lysis** ^∗^
	06-01750	ST228	No lysis
	06-00219	ST5	No lysis
	06-00409	ST225	No lysis

CA-MRSA	**03-02773**	**ST1**	**Full lysis**
	*06-00631*	*ST5*	*Partial lysis*
	*06-00373*	*ST8*	*Partial lysis*
	*05-01089*	*ST22*	*Partial lysis*
	06-02000	ST80	No lysis
	06-00467	ST152	No lysis

MRSA	South Transdanubian Regional Public Health Institute, Hungary (19 strains)		**Full lysis (8 strains)**
			No lysis (11 strains)
	N4315		No lysis
	MW2		No lysis
	MU3		No lysis
	MU50		No lysis
	S30		No lysis
	CI6005		No lysis
	Ci5734		No lysis
	116539		No lysis
	116550		No lysis

*S. aureus*	OEK 112016		No lysis
	OEK 112017		No lysis
	ATCC 29213		No lysis

## Data Availability

The data used to support the findings of this study are included within the article.
